# A Dysferlin Exon 32 Nonsense Mutant Mouse Model Shows Pathological Signs of Dysferlinopathy

**DOI:** 10.3390/biomedicines11051438

**Published:** 2023-05-13

**Authors:** Océane Ballouhey, Marie Chapoton, Benedicte Alary, Sébastien Courrier, Nathalie Da Silva, Martin Krahn, Nicolas Lévy, Noah Weisleder, Marc Bartoli

**Affiliations:** 1 Aix Marseille University, INSERM, MMG, U1251, 13005 Marseille, France; oceane.ballouhey@ac-aix-marseille.fr (O.B.); marie.chapoton@protonmail.com (M.C.); benedicte.alary@univ-amu.fr (B.A.); sebastien.courrier@univ-amu.fr (S.C.); nathalie.dasilva@univ-amu.fr (N.D.S.); martin.krahn@univ-amu.fr (M.K.); nicolas.levy@univ-amu.fr (N.L.); 2 Département de Génétique Médicale et de Biologie Cellulaire, AP-HM, Hôpital d’Enfants de la Timone, 13005 Marseille, France; 3 Department of Physiology & Cell Biology, Dorothy M. Davis Heart and Lung Research Institute, The Ohio State University Wexner Medical Center, Columbus, OH 43210, USA; noah.weisleder@osumc.edu

**Keywords:** dysferlin, LGMDR2, dystrophy

## Abstract

Dysferlinopathies are a group of autosomal recessive muscular dystrophies caused by pathogenic variants in the DYSF gene. While several animal models of dysferlinopathy have been developed, most of them involve major disruptions of the Dysf gene locus that are not optimal for studying human dysferlinopathy, which is often caused by single nucleotide substitutions. In this study, the authors describe a new murine model of dysferlinopathy that carries a nonsense mutation in Dysf exon 32, which has been identified in several patients with dysferlinopathy. This mouse model, called Dysf ^p.Y1159X/p.Y1159X^, displays several molecular, histological, and functional defects observed in dysferlinopathy patients and other published mouse models. This mutant mouse model is expected to be useful for testing various therapeutic approaches such as termination codon readthrough, pharmacological approaches, and exon skipping. Therefore, the data presented in this study strongly support the use of this animal model for the development of preclinical strategies for the treatment of dysferlinopathies.

## 1. Introduction

Locomotion and posture maintenance in animals require the contraction of skeletal muscle. Force constraints and mechanic stress/stretch may injure muscle tissue at the sarcolemmal level leading to the disruption of this cell membrane. Unrepaired lesions of the sarcolemma result in damage to the muscle fibres, leading eventually to their death. Therefore, efficient membrane repair after the disruption of the sarcolemma is essential for muscle cell survival. In several muscular disorders, this membrane repair response is non-functional which leads to muscle weakness and wasting. Dysferlinopathies, such as the Miyoshi myopathy (MM) and limb-girdle muscular dystrophy (LGMD) type 2B/R2, are characterized by muscle atrophy and weakness (DYSF, OMIM: 603009) [1,2]. Previously, LGMDR2 was distinguished from MM, as in LGMDR2 muscle weakness and atrophy were thought to begin in proximal muscles, while in MM muscle weakness was thought to begin mainly in the distal muscles. In fact, a recent assessment of both diseases has revealed that there is no clinical divergence in proximal vs. distal involvement.

The dysferlinopathies are autosomal recessive muscular dystrophies caused by mutations in the gene DYSF, encoding the protein dysferlin, a 230 kDa transmembrane protein that is highly expressed in skeletal muscle. Dysferlin is associated with the subsarcolemmal vesicles and sarcolemma, particularly within the transverse (T)-tubule invaginations of the sarcolemma. It has been demonstrated to be involved in several cellular functions such as protein vesicle trafficking [3], T-tubule formation [4], and the sarcolemma membrane repair process [5,6,7]. Mutations in DYSF can inactivate this sarcolemma membrane repair process, and the resulting cell death increases the degeneration/regeneration cycles of muscle, elevates inflammation, and causes extensive muscle fibrosis and fatty tissue infiltration [8,9]. Thus, patients with dysferlinopathies display elevated levels of creatine kinase in the blood serum, extensive muscle inflammation, and progressive muscle weakness [10]. Several treatments for dysferlinopathies and other muscular disorders are currently under development, some of which have shown promising results in clinical trials. However, despite several proof-of-principle studies on dysferlinopathy therapies such as gene therapy, exon skipping, and stop codon readthrough, there has been no clinical trial showing an effective treatment for dysferlinopathy patients [11,12,13]. 

Discovering new disease treatments or testing promising therapies benefits from having highly relevant animal models available. Most of the previously developed dysferlinopathy animal models display abnormal dysferlin expression due to genetic modifications that produce deletions or insertions in the Dysf locus (such as A/J, BLA/J or Dysftm1Kcam mice) [12,14,15]. Mice carrying a Dysf missense variant have also been described (such as SJL or MMex38 mice) [16,17,18]. There is extensive variability in the phenotype seen in these animals, particularly regarding the age at which pathology appears in skeletal muscles. For example, A/J mice show dystrophic hallmarks by histology at 4–5 months, whereas SJL mice show signs of pathology at 2 months. While there is variation in the age of onset in both models, the alterations at the histological level (variability of fibre diameters, centronucleation, inflammation, fibrosis, as well as degeneration and regeneration of myofibers) are present in both models. Given the limited number of genetic lesions that are modelled by the available mouse lines and the variability in age of onset of pathology, the field would benefit from the generation of additional mouse models of dysferlinopathy. 

Here, we report a mouse dysferlinopathy model harbouring a nonsense mutation found in dysferlinopathy patients, c.3477C > A (p.Y1159X) in exon 32 of the Dysf gene [19,20]. Our novel mouse model recapitulates the pathological mechanisms of dysferlinopathy making it an ideal model to test specific therapeutic approaches such as stop codon readthrough and other approaches to treat dysferlin deficiency. 

## 2. Materials and Methods

### 2.1. Mice

The mouse strain Dysf ^p.Y1159X/p.Y1159X^ was obtained from the CIML animal platform.

To create the Dysf ^p.Y1159X/p.Y1159X^ mouse model, we first cloned a 6.8 kb RP23-129H11 BAC containing exon 32 of the dysferlin gene. We then conducted directed mutagenesis to introduce a nonsense mutation into the vector: c.3477C > A (p.Y1159X). To excise the cassette coding for chloramphenicol resistance, we integrated a ClaI site on both primers. This cleavage resulted in two silent mutations at 23 bp from the desired mutation. Additionally, we added a new BgIII restriction site in exon 31, 200 bp upstream of the mutated exon 32. We used subsequent cloning to insert the ACN cassette, which contains the AsiSI site used to linearize the vector. This Neo cassette had resistance to neomycin and was used to select ES cells. The cassette was self-eliminated from the locus in heterozygous F1 mice, leaving behind an 85 bp sequence corresponding to a Lox site and some residual cloning sequences. This stand-alone cassette did not interfere with the expression of the dysferlin gene. Finally, we integrated a counter selection cassette upstream of exon 31, which was obtained from ES cells. ES cells that integrated the vector containing the mutation were sensitive to chloramphenicol, while those that did not integrate it or did not undergo homologous recombination as desired were antibiotic-resistant. After linearizing the vector through the site of the AsiSI restriction enzyme contained in the Neo cassette, we incorporated it by electroporation into recombinant ES cells. After confirming our vector integration in the ES cells, we microinjected them into C57BL/6 mice to generate chimeras. We then mated the chimeric males with WT females to obtain heterozygotes carrying the mutation, which resulted in the Dysf ^p.Y1159X/Y1159X^ strain.

Mice were maintained in a conventional animal facility with an enriched environment and a thermoregulated room with a 12 h light/12 h dark cycle. All experiments involving animals were performed in accordance with the European Directive for Animal Welfare (2010/63/EU) and received the French Ministry of Research authorization number APAFIS#26631-2020071713486143. Mice were genotyped using a standard method, a phenol/chloroform extraction of genomic DNA and then PCR with the following primers: Forward 5′-GCTTTCAAGCCAGTTGGGTC-3′ and Reverse 5′-TATGAGGAGCAGCTGCCCTG-3′. 

As the healthy control, the wild-type mouse strain C57BL/6N was obtained from Janvier laboratory. The mice were healthy and reproduced normally, and they did not differ significantly in their body mass. All mice analysed were male.

### 2.2. Genomic DNA Sequencing

Genomic DNA was extracted from a tail biopsy using phenol/chloroform extraction, and then Sanger sequencing of exon 32 and its intronic flanking region was performed using the following primers: Forward 5′-GTGTGTGTGTTGAGGCTCTAG-3′ and Reverse 5′-AGTGTGTGTAGCTGTGTAGTG-3′. 

### 2.3. Western Blot

Proteins were extracted from mice brachial biceps using RIPA buffer (Life technologies) and a protease inhibitor cocktail (Life technologies). Then, 40 µg of protein was loaded on each lane and separated by SDS-PAGE on 4–12% NuPAGE Bis-Tris gels (Life Technologies), using Chameleon Duo as a size marker, and transferred onto nitrocellulose membranes (at 100 V for 3 h at 4 °C). Membranes were blocked using fluorescent WB blocking buffer (Intercept blocking buffer, Li-Cor, ref 927-60001) in TBS 1× for 1 h at room temperature. Primary antibodies (Romeo (N-terminal epitope), 1:1000, abcam 124684) were then diluted in blocking buffer and incubated overnight at 4 °C. After washing in TBS-T, membranes were then incubated with secondary antibody (IRDye 680RD Donkey anti-goat, Li-Cor, ref 926-68074), and were diluted 1:10,000 in blocking buffer for 45 min at room temperature. The GAPDH (1:1000, abcam 9483) loading control was detected using a dilution of 1:10,000 of secondary antibodies (IRDye 680RD Donkey anti-rabbit, Li-Cor, ref 926-68073). The membranes were then washed in TBS-T and revealed using NIR Fluorescence LI-COR.

### 2.4. Histology and Immunohistochemistry

The tibialis anterior, quadriceps, and psoas muscles of mice were collected and sectioned, being of a 5 µm thickness, in cryostat. The abdominal fat biopsy was cut in microtome at 15 µm thickness. At least 10 mice for each genotype were analysed.

For haematoxylin eosin staining (HE), muscles and abdominal fat sections were fixed in 4% paraformaldehyde for 10 min and washed in PBS. Then sections were incubated in distilled water (30 s), in haematoxylin (2 min, Sigma HHS32, Saint-Quentin-Fallavier, France), in distilled water (30 s), in eosin (10 min, Sigma HT110232), and then were dehydrated with ethanol. Muscle sections were finally incubated for 1 min in xylene.

Sections from 18-month-old Dysf ^p.Y1159X/p.Y1159X^ quadriceps muscle were air-dried for 15 min, rinsed in 60% absolute ethanol for 5 min, and immersed in a double filtered solution of Oil Red O for 10 min. After a brief wash in distilled water, the sections were air dried and mounted.

Immunohistochemistry was performed on muscle sections using primary antibodies against dysferlin (Romeo, 1:200, abcam 124684) or aquaporin 4 (1:50, Santa Cruz sc-20812, Heidelberg, Germany). Primary antibodies were incubated for 2 h at room temperature. After washing in PBS-T, muscle sections were then incubated with secondary antibody (1:50, abcam 150073) for 1 h at room temperature.

For muscle fibre analysis, co-labelling with a primary anti-lamin A/C antibody (nucleus) (1:100, Santa Cruz sc-20681) and an anti-laminin α antibody (Extracellular Matrix) (1:300, abcam 11576) was carried out. A secondary HRP antibody was used for revelation (1/100, Invitrogen 31460 against anti-lamin A/C antibody, and Jackson 712-036-150 against anti-laminin α antibody). The fibre size and number were counted automatically, after manual verification, by the ellipse software. Nuclei and lobulations were counted using a semi-automated microscopic platform and Histolab software. This was performed for at least ten wild-type and Dysf ^p.Y1159X/p.Y1159X^ mice at 3, 5, 9, and 12 months.

### 2.5. Grip Test

The four limbs grip strength of wild-type and Dysf ^p.Y1159X/p.Y1159X^ mice was measured with the BIO-GS3 grip test (Bioseb, Vitrolles, France). Mice were placed on metal mesh with all four limbs and were slowly pulled back using the tail. The maximum tension was recorded, and the experiment was repeated 10 times for each mouse. This was performed for at least five wild-type mice and five Dysf ^p.Y1159X/p.Y1159X^ mice at 3, 9, and 12 months and four mice at 5 months.

### 2.6. Gait Analysis

Gait analysis was performed on wild-type and Dysf ^p.Y1159X/p.Y1159X^ mice at 3, 5, 9, and 12 months using a GaitLab (ViewPoint). The number of mice analysed was *n* = 6 for each condition, except at nine months the numbers were *n* = 4 for wild-type and *n* = 5 for Dysf ^p.Y1159X/p.Y1159X^. For each mouse, the experiment was repeated between 5 and 10 times. The software measured the four legs separately each time. The mice for each time point were trained twice a week for a month before the recordings.

The gait analysis was performed with 21 measurable parameters using GaitLab software (Table 1).

### 2.7. Statistical Analysis

For fibre area, centronucleation, lobulation, grip strength, and distance between the hind limbs, individual means were compared using the non-parametric Mann–Whitney test. Statistical powers of the tests were strictly superior at 84%. Differences were statistically significant if *p* < 0.05 (*) or if *p* < 0.01 (**). For gait analysis, we first calculated the reduced centred variable and then applied principal component analysis (PCA).

## 3. Results

**Figure 1:**
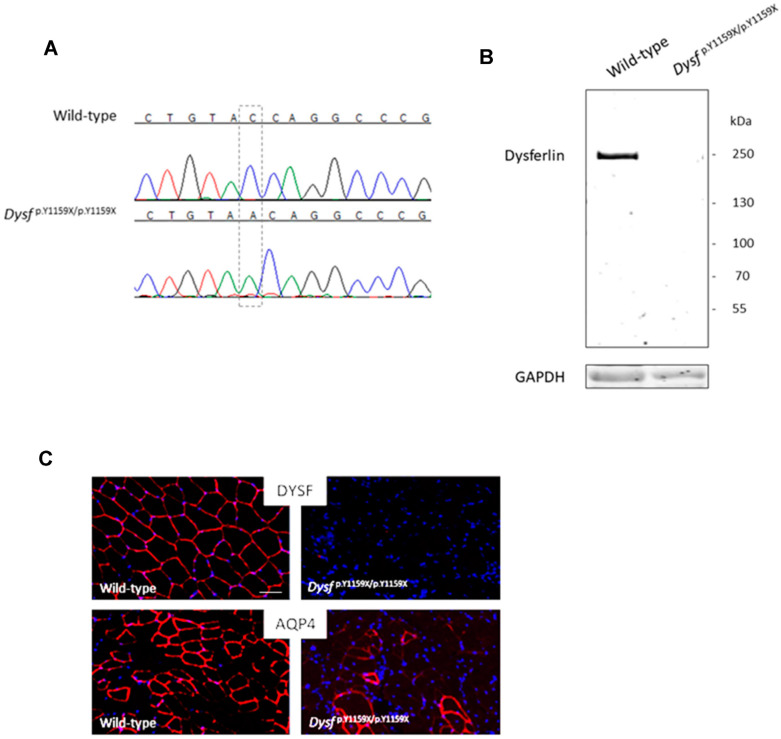
Dysf-nonsense mutant p.Y1159X leads to dysferlin absence in mice. (**A**) Chromatograms of DYSF exon 32 from wild-type and Dysf ^p.Y1159X/p.Y1159X^ mice. (**B**) Western blot analysis of dysferlin in the brachial biceps of wild-type and Dysf ^p.Y1159X/p.Y1159X^ mice. In total, 40 µg of protein was loaded on each lane and revealed with an antibody directed against the N-terminus of dysferlin (Hamlet-1). GAPDH was used for normalization. (**C**) Immuno-localization of dysferlin (DYSF, Romeo, red) and aquaporin 4 (AQP4, aquaporine-4, red) in tibialis anterior muscles from wild-type and Dysf ^p.Y1159X/p.Y1159X^ mice. DAPI was used as a nucleus marker (blue). No dysferlin signal was apparent in muscle section from Dysf ^p.Y1159X/p.Y1159X^ and drastically decreased in AQP-4 staining. Scale bar 50 µm.

To obtain the most appropriate mouse model to test the therapeutic strategies for patients carrying loss-of function pathogenic variants in the DYSF gene, we set out to generate a mouse line with a nonsense variant. We selected NM_003494.3: c.3477C > A (p.Y1159X) in exon 32 since it has been previously observed in three dysferlinopathy patients with a complete lack of dysferlin protein expression in muscle biopsies [18,19]. Moreover, since this variant is located in exon 32 of a 55-exon gene, nonsense-mediated decay (NMD) is predicted for this variant. Finally, even if the mutated DYSF transcript is not degraded, and a truncated protein is produced, it will not be functional since it will lack major functional domains located at the C-terminus. We created the mouse model Dysf ^p.Y1159X/p.Y1159X^ by integrating the cognate nonsense mutation c.3477C > A in exon 32 of the Dysf gene of C57BL/6 mice (Figure 1A). Our mouse model was generated using a targeting vector designed to replace a 6.8 kb region containing the mutation c.3477C > A. Similar to dysferlin expression results from patients carrying the c.3477C > A variant, a Western blot analysis of muscle tissue from Dysf ^p.Y1159X/p.Y1159X^ mice demonstrated the complete absence of dysferlin, both at the full-length size (250 kDa) and at the putative expected size for the truncated protein (140 kDa) (Figure 1B). The immunohistochemistry of Dysf ^p.Y1159X/p.Y1159X^ mice muscles confirmed the absence of dysferlin, and remarkably demonstrated a significant decrease in aquaporin 4 (AQP4) labelling (Figure 1C).

**Figure 2:**
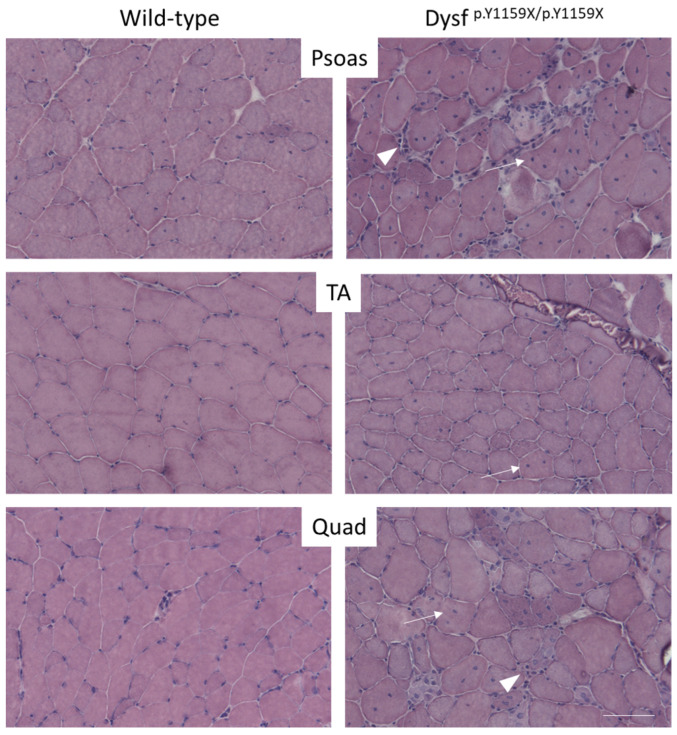
Dystrophic features are present in Dysf ^p.Y1159X/p.Y1159X^ mice. Histological analysis (HE staining) of psoas, tibialis anterior (TA), and quadriceps (Quad) muscles of Dysf ^p.Y1159X/p.Y1159X^ and wild-type mice at 5 months. Variable fibre size, centrally located nuclei (white thin arrows), and fibrosis (white thick arrows) are clearly visible in Dysf ^p.Y1159X/p.Y1159X^ muscles. Scale bar 100 µm.

To evaluate the impact of the introduced nonsense variant on muscle structure, we performed several histological analyses. The muscle section from Dysf ^p.Y1159X/p.Y1159X^ 5-month-old mice displayed many dystrophic features typically seen in patients affected by dysferlinopathies. The histological analysis of Dysf ^p.Y1159X/p.Y1159X^ mice muscles showed a dystrophic phenotype including variable fibre size, centronucleation, and fibrosis (Figure 2). 

**Figure 3:**
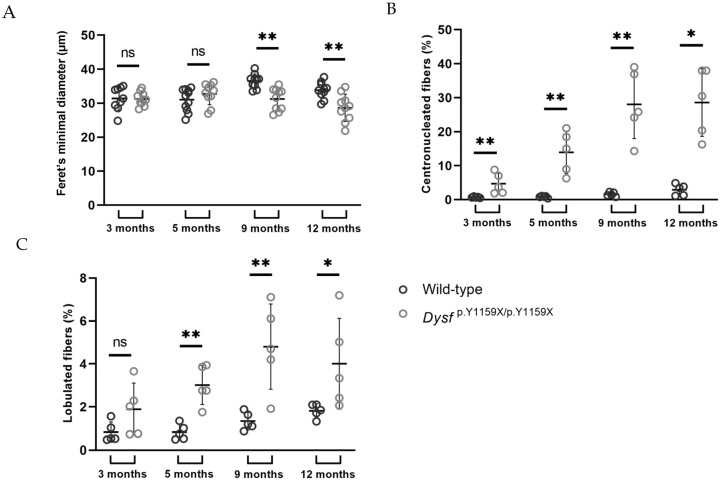
Quadriceps Dysf ^p.Y1159X/p.Y1159X^ fibres are smaller, more centronucleated and more lobulated than wild-type fibres. (**A**) Feret’s minimal diameters of wild-type and Dysf ^p.Y1159X/p.Y1159X^ mice quadriceps were measured using ellipse software at 3, 5, 9, and 12 months. Data are mean ± SD (standard deviation) for *n* = 10 mice. For each mouse, at least 790 fibres were analysed. Statistical analysis was performed with Mann–Whitney test (ns: non-significant; **: *p*-value < 0.01). (**B**) Centrally-located nuclei of wild-type and Dysf ^p.Y1159X/p.Y1159X^ mice quadriceps were quantified at 3, 5, 9, and 12 months. Data are mean ± SD (standard deviation) for *n* = 5 mice. For each mouse, at least 5300 fibres were analysed. Statistical analysis was performed with Mann–Whitney test (ns: non-significant; *: *p*-value < 0.05; **: *p*-value < 0.01). (**C**) Lobulated fibres of wild-type and Dysf ^p.Y1159X/p.Y1159X^ mice quadriceps were quantified at 3, 5, 9, and 12 months. Data are mean ± SD (standard deviation) for *n* = 5 mice. Statistical analysis was performed with Mann–Whitney test (ns: non-significant; *: *p*-value < 0.05; **: *p*-value < 0.01).

To quantify the changes in muscle tissues, fibre diameter was measured and centronucleated, and lobulated fibres were counted (Figure S1, Figure 2 and Figure 3). Starting at 9 months of age, the fibres of Dysf ^p.Y1159X/p.Y1159X^ mice were significantly smaller compared to wild-type mice (15% decrease at 9 months) (Figure 3A). There were significant increases in the central nucleus and lobulation from 3 months and 5 months of age, respectively (Figure 3B,C). Dysf ^p.Y1159X/p.Y1159X^ mice had between 6.5 and 17.5 times more centronucleated muscle fibres and between 2 and 3.5 times more lobulated muscle fibres compared to wild-type mice.

**Figure 4:**
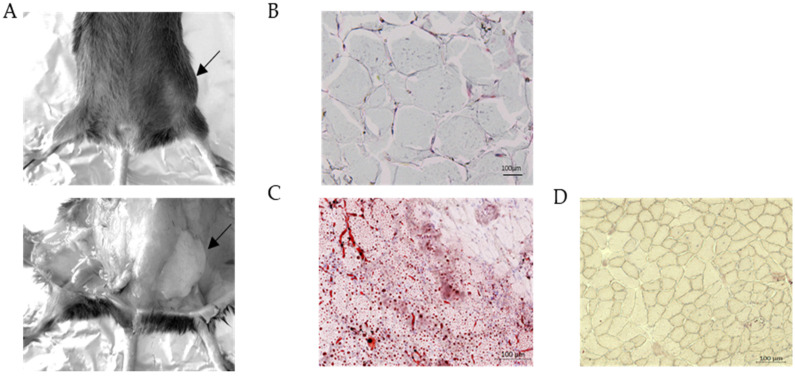
Accumulation of abdominal fat in old Dysf ^p.Y1159X/p.Y1159X^ mice. (**A**) Dissection and biopsy of abdominal fat of Dysf ^p.Y1159X/p.Y1159X^ mice at 18 months. Arrows indicate the localization of the fat. (**B**) Histological analysis of abdominal fat of Dysf ^p.Y1159X/p.Y1159X^ mice at 18 months. (**C**,**D**) Oil Red O staining of neutral lipid within skeletal muscle in a representative quadriceps muscle of Dysf ^pY1159X/pY1159X^ (**C**) and wild-type (**D**) mice at 18 months of age. Lipid droplets are viewed as red distinct spots.

There is an accumulation of subcutaneous abdominal fat in old Dysf ^p.Y1159X/p.Y1159X^ (Figure 4). These accumulations of abdominal fat are usually found on the flank or hip and appear to increase in size with age. These fat accumulations appear to be present in mice at 18 months of age. Histological analyses confirm that this mass corresponds to fat cells (Figure 4B), confirming that Dysf ^p.Y1159X/p.Y1159X^ mice have lipid accumulation abnormalities. Furthermore, when we analysed the muscle section from the quadriceps muscle at the same age via red oil staining, we observed numerous lipid droplets inside the myofibers in several parts of the muscle (Figure 4C).

**Figure 5:**
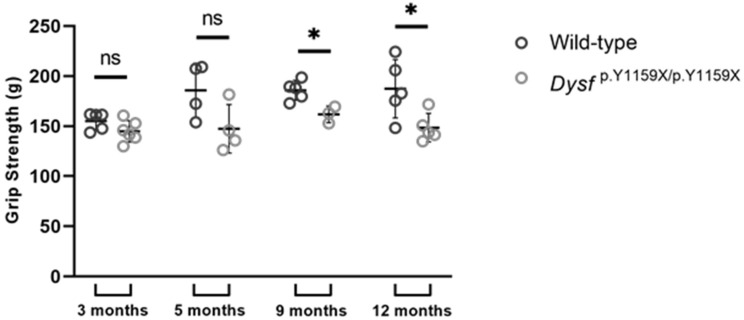
Dysf ^p.Y1159X/p.Y1159X^ mice have less strength than wild-type mice. Limb strengths of wild-type and Dysf ^p.Y1159X/p.Y1159X^ mice were measured using four grip tests at 3, 5, 9, and 12 months. Data are mean ± SD (standard deviation) for *n* = 5 mice except at 5 months *n* = 4 mice. For each mouse, at least 10 experiments were performed. Statistical analysis was performed with Mann–Whitney test (ns: non-significant; *: *p*-value < 0.05).

The major features of the phenotype of patients with dysferlinopathies are progressive degeneration and atrophy of skeletal muscles leading to muscle weakness. To explore the muscle strengths of mice, four limb grip tests were performed for at least four wild-type mice and four Dysf ^p.Y1159X/p.Y1159X^ mice at 3, 5, 9, and 12 months. From 9 months of age, a significant decrease in muscle strength was observed in Dysf ^p.Y1159X/p.Y1159X^ mice compared to wild-type mice (Figure 5). At 3 months of age, the strength of Dysf ^p.Y1159X/p.Y1159X^ mice was 6.6% lower than that of wild-type mice. This decrease in strength was more pronounced with age, reaching a 20% lower strength at 12 months old in Dysf ^p.Y1159X/p.Y1159X^ compared to wild-type mice.

**Figure 6:**
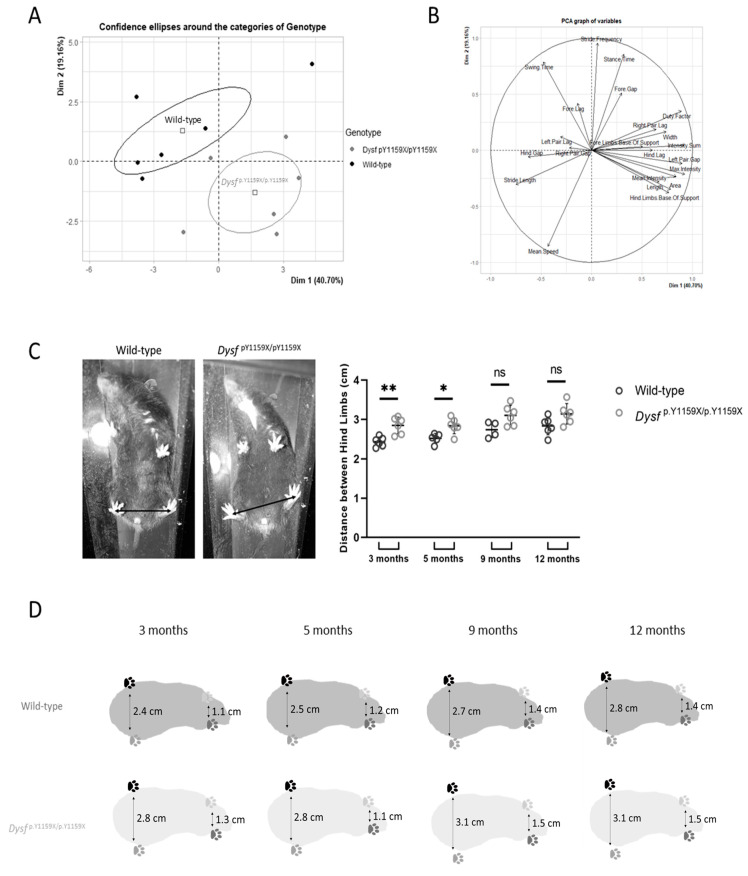
Gait analysis shows differences between wild-type mice and Dysf ^p.Y1159X/p.Y1159X^ mice. (**A**) Linear discriminant analysis of gait parameters for wild-type and Dysf ^p.Y1159X/p.Y1159X^ mice at 12 months. Individual measurements (dots) and groups (95% confidence ellipses) are placed. (**B**) Factor chart of Dim1 and Dim2 variables. (**C**) Determination of the distance between hind limbs of wild-type and Dysf ^p.Y1159X/p.Y1159X^ mice at 3, 5, 9, and 12 months. Data are mean ± SD (standard deviation) for *n* = 6 mice, except *n* = 4 for wild-type mice at 9 months. For each mouse, at least 5 gait tests were performed. Statistical analysis was performed with Mann–Whitney test (ns: non-significant; *: *p*-value < 0.05; **: *p*-value < 0.01). (**D**) Evolution of distance between hind and fore limbs with age.

Next, we explored the gait of the Dysf ^p.Y1159X/p.Y1159X^ mice for at least 10 wild-type and Dysf ^p.Y1159X/p.Y1159X^ mice at 3, 5, 9, and 12 months. The gait analysis of a mouse was performed using 21 measurable parameters including the distance between hind or fore limbs, mean speed, and stride frequency (Table 1). First, we reduced the large data set by principal component analysis (PCA) to decide on the most relevant parameters. We analysed differences between wild-type and Dysf ^p.Y1159X/p.Y1159X^ mice at 3, 5, 9, and 12 months. At 12 months of age, the extracted 21 components described 59.86% of the total variance (40.70% for the 1st and 29.76% for the 2nd dimension), characterizing different aspects of gait between wild-type and Dysf ^p.Y1159X/p.Y1159X^ mice (Figure 6A).

Ellipses drawn around clusters identified after automatic classification confirmed gait differences between wild-type and Dysf ^p.Y1159X/p.Y1159X^ mice within 95% confidence intervals (Figure 6A). We investigated the components that accounted for the most variation and hence were the most likely to expose differences between the mice genotypes. Eleven components were characterized according to the highest loadings (>0.75), which can be described as the correlation coefficient between the parameter and component (Figure 6B). The 11 extracted gait parameters can be attributed to different dimensions of gait performance, such as spatial and temporal aspects. Besides the speed of the mice, the distance between limbs showed the most significant difference between wild-type and Dysf ^p.Y1159X/p.Y1159X^ mice starting at 3 months of age (Figure 6C). At the ages of 3 and 5 months, a significant increase in distance between hind limbs was measured in Dysf ^p.Y1159X/p.Y1159X^ compared to wild-type mice: a 17% increase in 3-month-old mice and a 13% increase in 5-month-old mice. Similarly, the distance between the fore limbs of Dysf ^p.Y1159X/p.Y1159X^ mice was greater than that of wild-type mice: from 7% to 18% increase in distance between fore limbs (Figure 6D). These results showed that Dysf ^p.Y1159X/p.Y1159X^ mice have difficulties supporting their weight, and thus their hind and fore limbs are spread apart.

## 4. Discussion

To study the pathological mechanisms leading to dysferlinopathies and to investigate therapeutic strategies, we created the mouse model Dysf ^p.Y1159X/p.Y1159X^ by integrating the nonsense mutation NM_003494.3: c.3477C > A (p.Y1159X) which leads to dysferlin absence. This pathogenic variant has been identified in three patients affected with dysferlinopathy, characterized by muscle weakness and muscle dystrophic pattern. The nonsense mutation integrated in Dysf ^p.Y1159X/p.Y1159X^ mice caused a similar phenotype including muscle damage, loss of muscle strength, and gait changes in mice. Indeed, as for patients affected with dysferlinopathies, a histological analysis of Dysf ^p.Y1159X/p.Y1159X^ mice muscles showed a dystrophic phenotype, including variable fibre size diameter, centronucleation, and fibrosis. These dystrophic features were also found in other dysferlin-deficient mice models that showed degenerating and regenerating fibres with centrally located nuclei and marked variations in fibre diameter [14]. Moreover, these dystrophic features were identified earlier in our mouse model than in SJL mice where it appears at 2–3 months of age. This indicates that before the symptoms appear, the function of muscle is already affected and starts to degenerate.

Dysferlinopathies are also characterized by lipid accumulations within muscle fibres and abnormalities of lipid metabolism. In our mouse model, we confirmed that the quadriceps muscle from Dysf ^p.Y1159X/p.Y1159X^ at 18 months of age presented with lipid droplets within the myofibers. Our analyses also showed a clear presence of abdominal fat accumulation in old Dysf ^p.Y1159X/p.Y1159X^ mice. These results were consistent with the observations made in patients with muscular dystrophies [8,9] and in a previous dysferlin-deficient mouse model [21,22]. The fat accumulations in the hips of the mice do not have a key role in the pathological mechanism of dysferlinopathies, but they suggest the presence of a metabolic response due to the sedentary nature of Dysf ^p.Y1159X/p.Y1159X^ mice. Indeed, the loss of muscular strength eventually leads to less activity, which may trigger a metabolic response that results in fat storage. Moreover, the presence of a lipid droplet within the muscle is a clear sign of an advanced dystrophic muscle. 

Our analyses also showed a significant decrease in aquaporin 4 protein levels in the muscle fibres of Dysf ^p.Y1159X/p.Y1159X^ mice compared to wild-type mice. Aquaporin 4 is a protein located in the sarcolemma and specializes in the permeability of the membrane to water [23]. In line with our results, one study had previously demonstrated a reduction between 23% to 95% in aquaporin 4 in six patients affected with dysferlinopathies [24]. In this study, immunoprecipitation experiments did not show any direct interaction between dysferlin and aquaporin 4 (data not shown), suggesting that dysferlin deficiency may lead to a downregulation of aquaporin 4 levels. These results suggest that aquaporin 4 could be a biomarker in dysferlinopathies and may serve useful for the evaluation of the effectiveness of therapeutic strategies.

The nonsense-mutation-induced, dysferlin-deficient mice model showed a decrease in muscle strength. Interestingly, muscle strength was not affected in SJL/J mice, another dysferlin-deficient model [17]. However, this study was conducted in mice from 9 weeks to 25 weeks of age, and our results indicated that the decrease in muscle strength in Dysf ^p.Y1159X/p.Y1159X^ mice was significant from the age of 9 months. This decrease in muscle strength intensified with age, reaching a 20% lower strength in Dysf ^p.Y1159X/p.Y1159X^ mice compared to wild-type mice at 12 months. These results showed progressive muscle weakness in all four limbs of Dysf ^p.Y1159X/p.Y1159X^ mice. This progressive muscle weakness is known and documented in patients with dysferlinopathies [25]. It is therefore essential to better characterize the pathology mechanisms of dysferlinopathies in order to carry out long-term phenotypic studies in dysferlin-deficient animal models.

Patients affected with dysferlinopathies have gait abnormalities and difficulty moving due to muscle weakness [26,27]. The gait analysis in the dysferlin-deficient mouse model thus makes it possible to bring new data to the dysferlinopathies phenotype. Our results of gait analysis indicated a clear difference between the gait of Dysf ^p.Y1159X/p.Y1159X^ mice and the gait of wild-type mice, which is consistent with the phenotype of dysferlinopathies patients who have gait abnormalities. Moreover, gait analysis demonstrated an increase of at least 7% in the distance between fore limbs and at least 11% in the distance between the hind limbs of Dysf ^p.Y1159X/p.Y1159X^ mice compared to wild-type mice. These observations were consistent with the results of the grip test and confirmed the presence of proximal muscle weakness in all four limbs of Dysf ^p.Y1159X/p.Y1159X^ mice. Moreover, these results show that postural muscles are mainly impacted by dysferlin deficiency. 

## 5. Conclusions

In conclusion, our mouse model Dysf ^p.Y1159X/p.Y1159X^ recapitulated most aspects of the phenotype of previous dysferlin-deficient mouse models and dysferlinopathy patients. Dysf ^p.Y1159X/p.Y1159X^ mice presented variable muscle fibre sizes, centrally located nuclei, fibrosis, lobulated muscle fibres, a loss of muscle strength, gait abnormalities, and lipid abnormalities. However, it is important to note that Dysf ^p.Y1159X/p.Y1159X^ mice had a moderate phenotype since dysferlin absence did not affect overall mobility, reproduction, or the lifespan of mice. This new mouse model can be used to decipher the pathological mechanism of dysferlinopathies, but also, more specifically, to investigate therapeutic strategies such as gene therapy or pharmacological approaches. 

## Figures and Tables

**Table 1:** This table presents the 21 parameters measured to quantify the gait from the GaitLab software.

Parameters	Unit	Description
Lag	s	time difference between front or hind limbs
Gap	cm	distance between front or hind limbs
Pair Lag	s	time difference between limbs on the same side
Even gap	cm	distance between limbs on the same side
Limbs Base of Support	cm	distance between the two hind limbs or front limbs
Mean speed	cm/s	-
Stride frequency	s	time for a limb on the ground and in the air
Stance time	s	time for a limb on the ground
Swing time	s	time for a limb in the air
Stride length	cm	distance between the placement of the same limb
Duty factor	-	ratio of the time of use of a limb to the total running time
Area	cm^2^	area of the animal paw
Max intensity	grey level	max pressure that the animal applies on its paw when it walks
Mean intensity	grey level	mean pressure that the animal applies on its paw when it walks
Width	cm	width of the animal paw
Length	cm	length of the animal paw

## Data Availability

The data presented in this study are available on request from the corresponding authors.

## Supplementary Materials

The following supporting information can be downloaded at: https://www.mdpi.com/article/10.3390/biomedicines11051438/s1. Figure S1: Representative images of muscle tissues that were used to measure the Feret’s minimal diameter of the fibers, the number of centronucleated fibers, and the degree of fiber lobulation. To visualize the muscle tissue, we performed staining with two different antibodies: anti-lamin A/C for labeling the nucleus and anti-laminin α for delimiting the sarcolemma.

## References

[1] Bashir R., Britton S., Strachan T., Keers S., Vafiadaki E., Lako M., Richard I., Marchand S., Bourg N., Argov Z. (1998). A gene related to Caenorhabditis elegans spermatogenesis factor fer-1 is mutated in limb-girdle muscular dystrophy type 2B. Nat. Genet..

[2] Liu J., Aoki M., Illa I., Wu C., Fardeau M., Angelini C., Serrano C., Urtizberea J.A., Hentati F., Ben Hamida M. (1998). Dysferlin, a novel skeletal muscle gene, is mutated in Miyoshi myopathy and limb girdle muscular dystrophy. Nat. Genet..

[3] Lek A., Evesson F.J., Sutton R.B., North K.N., Cooper S.T. (2012). Ferlins: Regulators of Vesicle Fusion for Auditory Neurotransmission, Receptor Trafficking and Membrane Repair. Traffic.

[4] Demonbreun A.R., Rossi A.E., Alvarez M.G., Swanson K.E., Deveaux H.K., Earley J.U., Hadhazy M., Vohra R., Walter G.A., Pytel P. (2014). Dysferlin and Myoferlin Regulate Transverse Tubule Formation and Glycerol Sensitivity. Am. J. Pathol..

[5] Bansal D., Miyake K., Vogel S.S., Groh S., Chen C.-C., Williamson R., McNeil P.L., Campbell K.P. (2003). Defective membrane repair in dysferlin-deficient muscular dystrophy. Nature.

[6] Defour A., Van der Meulen J.H., Bhat R., Bigot A., Bashir R., Nagaraju K., Jaiswal J.K. (2014). Dysferlin regulates cell membrane repair by facilitating injury-triggered acid sphingomyelinase secretion. Cell Death Dis..

[7] Lek A., Evesson F.J., Lemckert F.A., Redpath G.M.I., Lueders A.-K., Turnbull L., Whitchurch C.B., North K.N., Cooper S.T. (2013). Calpains, Cleaved Mini-Dysferlin_C72_, and L-Type Channels Underpin Calcium-Dependent Muscle Membrane Repair. J. Neurosci..

[8] Srivastava N.K., Yadav R., Mukherjee S., Pal L., Sinha N. (2017). Abnormal lipid metabolism in skeletal muscle tissue of patients with muscular dystrophy: In vitro, high-resolution NMR spectroscopy based observation in early phase of the disease. Magn. Reson. Imaging.

[9] White Z., Hakim C.H., Theret M., Yang N.N., Rossi F., Cox D., Francis G.A., Straub V., Selby K., Panagiotopoulos C. (2020). High prevalence of plasma lipid abnormalities in human and canine Duchenne and Becker muscular dystrophies depicts a new type of primary genetic dyslipidemia. J. Clin. Lipidol..

[10] Fanin M., Angelini C. (2002). Muscle pathology in dysferlin deficiency. Neuropathol. Appl. Neurobiol..

[11] Krahn M., Wein N., Bartoli M., Lostal W., Courrier S., Bourg-Alibert N., Nguyen K., Vial C., Streichenberger N., Labelle V. (2010). A Naturally Occurring Human Minidysferlin Protein Repairs Sarcolemmal Lesions in a Mouse Model of Dysferlinopathy. Sci. Transl. Med..

[12] Lostal W., Bartoli M., Bourg N., Roudaut C., Bentaïb A., Miyake K., Guerchet N., Fougerousse F., McNeil P., Richard I. (2010). Efficient recovery of dysferlin deficiency by dual adeno-associated vector-mediated gene transfer. Hum. Mol. Genet..

[13] Barthélémy F., Blouin C., Wein N., Mouly V., Courrier S., Dionnet E., Kergourlay V., Mathieu Y., Garcia L., Butler-Browne G. (2015). Exon 32 Skipping of Dysferlin Rescues Membrane Repair in Patients’ Cells. J. Neuromuscul. Dis..

[14] Ho M., Post C.M., Donahue L.R., Lidov H.G.W., Bronson R.T., Goolsby H., Watkins S.C., Cox G.A., Brown R.H. (2004). Disruption of muscle membrane and phenotype divergence in two novel mouse models of dysferlin deficiency. Hum. Mol. Genet..

[15] Wiktorowicz T., Kinter J., Kobuke K., Campbell K.P., Sinnreich M. (2015). Genetic characterization and improved genotyping of the dysferlin-deficient mouse strain Dysf tm1Kcam. Skelet. Muscle.

[16] Bittner R.E., Anderson L.V., Burkhardt E., Bashir R., Vafiadaki E., Ivanova S., Raffelsberger T., Maerk I., Höger H., Jung M. (1999). Dysferlin deletion in SJL mice (SJL-Dysf) defines a natural model for limb girdle muscular dystrophy 2B. Nat. Genet..

[17] Rayavarapu S., Van Der Meulen J.H., Gordish-Dressman H., Hoffman E., Nagaraju K., Knoblach S.M. (2010). Characterization of Dysferlin Deficient SJL/J Mice to Assess Preclinical Drug Efficacy: Fasudil Exacerbates Muscle Disease Phenotype. PLoS ONE.

[18] Malcher J., Heidt L., Goyenvalle A., Escobar H., Marg A., Beley C., Benchaouir R., Bader M., Spuler S., García L. (2018). Exon Skipping in a Dysf-Missense Mutant Mouse Model. Mol. Ther. Nucleic Acids.

[19] Nguyen K., Bassez G., Bernard R., Krahn M., Labelle V., Figarella-Branger D., Pouget J., Hammouda E.H., Béroud C., Urtizberea A. (2005). Dysferlin mutations in LGMD2B, Miyoshi myopathy, and atypical dysferlinopathies. Hum. Mutat..

[20] Krahn M., Béroud C., Labelle V., Nguyen K., Bernard R., Bassez G., Figarella-Branger D., Fernandez C., Bouvenot J., Richard I. (2009). Analysis of the *DYSF* mutational spectrum in a large cohort of patients. Hum. Mutat..

[21] Grounds M.D., Terrill J.R., Radley-Crabb H.G., Robertson T., Papadimitriou J., Spuler S., Shavlakadze T. (2014). Lipid Accumulation in Dysferlin-Deficient Muscles. Am. J. Pathol..

[22] Haynes V.R., Keenan S., Bayliss J., Lloyd E.M., Meikle P., Grounds M.D., Watt M.J. (2019). Dysferlin deficiency alters lipid metabolism and remodels the skeletal muscle lipidome in mice. J. Lipid Res..

[23] Frigeri A., Nicchia G.P., Balena R., Nico B., Svelto M. (2004). Aquaporins in skeletal muscle: Reassessment of the functional role of aquaporin-4. FASEB J..

[24] Assereto S., Mastrototaro M., Stringara S., Gazzerro E., Broda P., Nicchia G.P., Svelto M., Bruno C., Nigro V., Lisanti M. (2008). Aquaporin-4 expression is severely reduced in human sarcoglycanopathies and dysferlinopathies. Cell Cycle.

[25] Harris E., Bladen C.L., Mayhew A., James M., Bettinson K., Moore U., Smith F.E., Rufibach L., Cnaan A., Bharucha-Goebel D.X. (2016). The Clinical Outcome Study for dysferlinopathy. Neurol. Genet..

[26] Mahjneh I., Marconi G., Bushby K., Anderson L.V., Tolvanen-Mahjneh H., Somer H. (2001). Dysferlinopathy (LGMD2B): A 23-year follow-up study of 10 patients homozygous for the same frameshifting dysferlin mutations. Neuromuscul. Disord..

[27] Angelini C., Peterle E., Gaiani A., Bortolussi L., Borsato C. (2011). Dysferlinopathy course and sportive activity: Clues for possible treatment. Acta Myol. Myopathies Cardiomyopathies Off. J. Mediterr. Soc. Myol..

