# Supplementary material for: A Dysferlin Exon 32 Nonsense Mutant Mouse Model Shows Pathological Signs of Dysferlinopathy
Source: Biomedicines. 2023 May 13;11(5):1438. doi: 10.3390/biomedicines11051438 (PMC10216094; doi:10.3390/biomedicines11051438)
Supplement: Supplementary file 1 [file biomedicines-11-01438-s001.zip › biomedicines-2351359-supplementary.pdf]

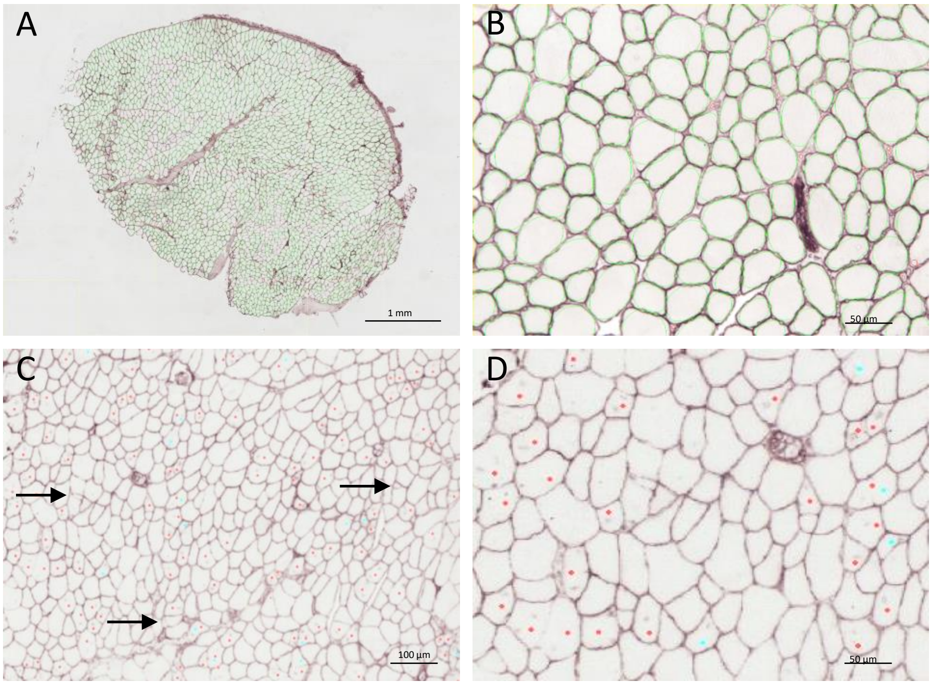

Figure Supp S1 : Representative images of muscle tissues that were used to measure the Feret's minimal diameter of the fibers, the number of centronucleated fibers, and the degree of fiber lobulation. To visualize the muscle tissue, we performed staining with two different antibodies: anti-lamin A/C for labeling the nucleus and anti-laminin α for delimiting the sarcolemma. The resulting images (panels **A, B, C, and D**) reveal the structure of the muscle tissues.

To quantify the size and number of muscle fibers, we utilized the Ellipse software, an automated counting software. This software generated green ellipses around the fibers, which were then verified manually to ensure the accuracy of the measurements. An example of the software output is shown in panel (**B)**, which displays a zoomed-in image of a muscle section. To quantify the number of centronucleated fibers, we used a different approach. We counted the number of centrally localized nuclei, which appear as red dots in panel (**C)**. This task required manual counting, but it was facilitated by the clear staining of the nuclei with anti-lamin A/C antibody. An example of the manual counting process is shown in panel **D**, which displays a zoomed-in image of a muscle section. The red dots indicate the centrally localized nuclei, which are characteristic of regenerating fibers.

Finally, to quantify the degree of fiber lobulation, we used a semi-automated microscopic platform and Histolab software. This approach allowed us to count the number of lobulations per muscle, which are indicated by arrows in panel (**C)**.
